# Editorial: Achievement Emotions in University Teaching and Learning, Students' Stress and Well-being

**DOI:** 10.3389/fpsyg.2022.910980

**Published:** 2022-04-27

**Authors:** Jesús de la Fuente, Douglas F. Kauffman, Meryem Yilmaz Soylu

**Affiliations:** ^1^School of Education and Psychology, University of Navarra, Pamplona, Spain; ^2^Department of Psychology, School of Psychology, University of Almería, Almería, Spain; ^3^Medical University of the Americas, Devens, MA, United States; ^4^Georgia Institute of Technology, Atlanta, GA, United States

**Keywords:** achievement emotions, learning and teaching, university, stress, wellbeing

Current research in achievement emotions, as a topic in Educational Psychology, has meant a paradigm shift, broadening the research panorama to include different motivational-affective variables, going beyond the prevailing research paradigm with an exclusively cognitivism-based focus. It has therefore stimulated analysis and inquiry into different issues which had not formerly been analyzed with rigor. Six manuscripts have analyzed this problem, providing diverse evidence of the different relationships: Cognitive Test Anxiety, Motivation, and Self-Regulation Through Curvilinear Analyses (Cassady and Finch), Mental Health and Academic Performance (Dekker et al.), Coping Strategies and Self-Efficacy (Freire et al.), Philosophical Inquiry and Students Engage in Learning (Leng), the Perception of Support in the Classroom and students' Motivation and Emotions (Trigueros et al.), and the resilience and creativity (Fan et al.).

On the one hand, current research now addresses meta-motivational and meta-affective processes, through analyzing the effects of achievement emotions on classic cognitive processes of learning. On the other hand, this domain has helped point research into the role of individual differences in the achievement emotions experienced, based on how they relate to powerful, classic variables of personality and cognition. Three research reports have analyzed these relationships between some of them: the relationship with emotional problems and adaptation to the university, in cyberbullying (Martínez-Monteagudo et al.), the preventing stress among undergraduate learners, and the importance of emotional intelligence, resilience, and emotion regulation (Thomas and Zolkoski), and the role of active coping in the relationship between learning burnout and sleep quality among college students (Wang et al.).

Moreover, the analysis of achievement emotions is being contextualized within academic teaching-learning contexts, where these emotions are commonly produced, so that they can be assessed and improved. In addition, this research paradigm does not overlook the importance of explanatory, predictive models of students' wellbeing and their psychological health, given that the university context is highly predictive of academic stress. Six manuscripts have provided evidence regarding the relevance of the teaching-learning process in various variables, based on the *Self-vs External- Regulated Learning Theory*: regarding achievement emotions (de la Fuente, Martínez-Vicente et al.), strategies for coping with academic stress (de la Fuente, Amate et al.), the factors and symptoms of academic Stress (de la Fuente, Peralta-Sánchez et al.), the learning approaches, academic achievement, and satisfaction (de la Fuente, Sander, Kauffmann and Yilmaz Soylu, 2020), and academic behavioral confidence and procrastination (de la Fuente, Sander, Garzón-Umerenkova et al.).

In conclusion, in this Research Topic was presented theoretical and empirical-based studies, and evidence-based proposals. The submitted manuscripts aim to minimize university students' experience of stress and to promote their wellbeing and psychological/emotional health through psychological assessment and intervention.

## Author Contributions

All authors listed have made a substantial, direct, and intellectual contribution to the work and approved it for publication.

## Funding

This work was supported by R&D Project PGC2018-094672-B-I00, University of Navarra (Ministry of Science and Education, Spain) and R&D Project UAL18 SEJ-DO31-A-FEDER, University of Almería, Spain (European Social Fund).

## Conflict of Interest

The authors declare that the research was conducted in the absence of any commercial or financial relationships that could be construed as a potential conflict of interest.

## Publisher's Note

All claims expressed in this article are solely those of the authors and do not necessarily represent those of their affiliated organizations, or those of the publisher, the editors and the reviewers. Any product that may be evaluated in this article, or claim that may be made by its manufacturer, is not guaranteed or endorsed by the publisher.

